# Electrically and Thermally Conductive Carbon Fibre Fabric Reinforced Polymer Composites Based on Nanocarbons and an *In-situ* Polymerizable Cyclic Oligoester

**DOI:** 10.1038/s41598-018-25965-w

**Published:** 2018-05-16

**Authors:** Ji-un Jang, Hyeong Cheol Park, Hun Su Lee, Myung-Seob Khil, Seong Yun Kim

**Affiliations:** 1 Mutifunctional Structural Composite Research Center, Institute of Advanced Composite Materials, Korea Institute of Science and Technology (KIST), 92 Chudong-ro, Bongdong-eup, Wanju-gun, Jeonbuk 55324 Republic of Korea; 2 Department of Organic Materials and Fiber Engineering, Chonbuk National University, 567 Baekje-daero, Jeonju-si, Jeonbuk 54896 Republic of Korea

## Abstract

There is growing interest in carbon fibre fabric reinforced polymer (CFRP) composites based on a thermoplastic matrix, which is easy to rapidly produce, repair or recycle. To expand the applications of thermoplastic CFRP composites, we propose a process for fabricating conductive CFRP composites with improved electrical and thermal conductivities using an *in-situ* polymerizable and thermoplastic cyclic butylene terephthalate oligomer matrix, which can induce good impregnation of carbon fibres and a high dispersion of nanocarbon fillers. Under optimal processing conditions, the surface resistivity below the order of 10^+10^ Ω/sq, which can enable electrostatic powder painting application for automotive outer panels, can be induced with a low nanofiller content of 1 wt%. Furthermore, CFRP composites containing 20 wt% graphene nanoplatelets (GNPs) were found to exhibit an excellent thermal conductivity of 13.7 W/m·K. Incorporating multi-walled carbon nanotubes into CFRP composites is more advantageous for improving electrical conductivity, whereas incorporating GNPs is more beneficial for enhancing thermal conductivity. It is possible to fabricate the developed thermoplastic CFRP composites within 2 min. The proposed composites have sufficient potential for use in automotive outer panels, engine blocks and other mechanical components that require conductive characteristics.

## Introduction

Carbon fibre reinforced polymer (CFRP) composites exhibit excellent mechanical properties that are comparable to those of structural metals and are much lighter in weight^1–3^. For this reason, CFRP composites are widely used as lightweight structural materials, especially for aircraft. For example, carbon fibre (CF) laminates or sandwiches are responsible for more than 50 wt% of the recently released Boeing 787 Dreamliner aircraft^4^. Despite their excellent performance, the CFRP composites are not in active use for automotive weight reduction because they are difficult to put into rapid production based on process automation, which is required in the automotive industry^5^. BMW has succeeded in applying a high-pressure resin transfer moulding process to the automated rapid production of lightweight composite components, which are intended for achieving weight reductions for its electric car models^5,6^. However, because of the potential difficulties with repairing or recycling CFRP composites that are based on a thermosetting epoxy matrix, there is much interest in CFRP composites that are fabricated using easily repairable and recyclable thermoplastic resins^7^.

When engineered thermoplastics are applied to CFRP composites, the high melt viscosity of thermoplastic resins may cause a critical problem by keeping the continuous carbon structures from being impregnated^7,8^. A recent method proposed to make up for this problem by using the ring-opening, polymerizable and low-viscosity cyclic butylene terephthalate (CBT) resin, which is a macrocyclic oligomer, for the fabrication of thermoplastic CFRP composites^9,10^. When heated above 150 °C, CBT molecules melt and impregnate CF fabrics with a low viscosity of 0.02 Pa·s. With further heating above 170 °C, they polymerize to form thermoplastic CFRP composites^7–9^. Among the various macrocyclic oligomers, CBT oligomers are promising because of their low viscosity, good mechanical properties and usefulness in various applications^8^.

CFRP composites, which are based on low-viscosity macrocyclic oligomers, show excellent mechanical properties; however, their applications are limited because of their limited electrical and thermal conductivities. In this context, we can expect that the application of these composites will be expanded to the development of thermoplastic CFRP composites with excellent conductivities. Unlike in percolation theory, where the electrical properties of composites are thought to rapidly increase when there is a certain amount of filler content due to the tunnelling effect of electrons^11–13^, there is an increasing monotonous relationship between the filler content and the thermal conductivity of composites^14–18^. Accordingly, when composites of high heat dissipation are manufactured, the composites should be highly filled with thermally conductive fillers; consequently, the manufacturing process will face substantial limitations. In this sense, there is a need to develop a new process that allows CFRP composites to be filled with high-volume content of thermally conductive fillers and that keeps the fillers uniformly dispersed.

**Figure 1:**
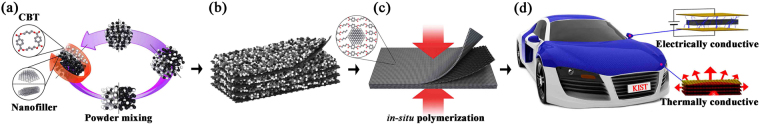
Schematic of the proposed process for fabricating rapidly-producible, repairable, and recyclable CFRP composites with electrically and thermally conductive characteristics.

To fabricate rapidly producible, repairable, and recyclable CFRP composites with excellent conductivities, as shown in Fig. 1, this paper proposes a new fabrication process for composites, which is based on powder mixing and *in-situ* polymerization of CBT oligomers. This process allows for a high content of fillers and their excellent dispersion, while also allowing for a good impregnation of CF fabrics with the thermoplastic resin. To optimize the conductive and mechanical properties of CFRP composites, nanocarbon fillers with excellent conductivities were incorporated into the CFRP composites by using the proposed fabrication process. Simultaneous improvements in the mechanical and conductive properties of the three-component composites were investigated and discussed.

## Results and Discussion

### Internal Structure

**Figure 2:**
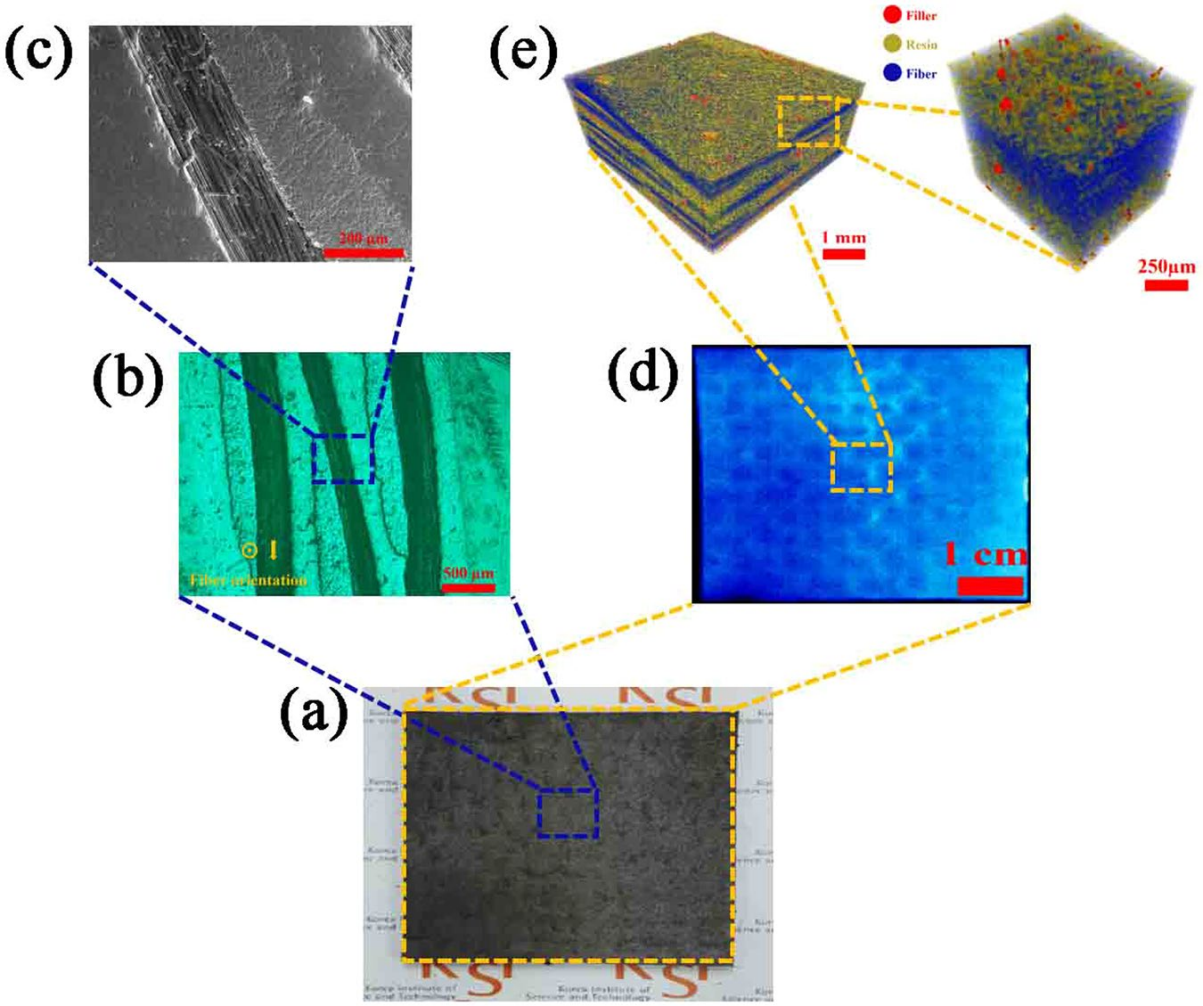
Analysis for internal structure of CFRP composites: (**a**) fabricated CFRP specimen, (**b**) OM to observe surface pore/defect, CF fabric impregnation and CF orientation, (**c**) FE-SEM to investigate nanofiller dispersion, surface pore/defect, CF fabric impregnation and CF orientation, (**d**) active thermography for measuring internal defects (>6 μm) on the entire area of the specimen, and (**e**) micro-CT to evaluate 3D realistic internal structure including nanofiller dispersion, internal defects (>0.7 μm) and CF architecture.

Nanocarbon filler-incorporated CFRP composites require accurate internal structure analysis for nanofiller dispersion, CF fabric impregnation, CF orientation and pore/defect evaluation. In this study, various tools, such as an optical microscope (OM), a field emission scanning electron microscope (FE-SEM), active thermography and X-ray micro-computed tomography (micro-CT), were applied for accurate internal structure analysis, as shown in Fig. 2.

**Figure 3:**
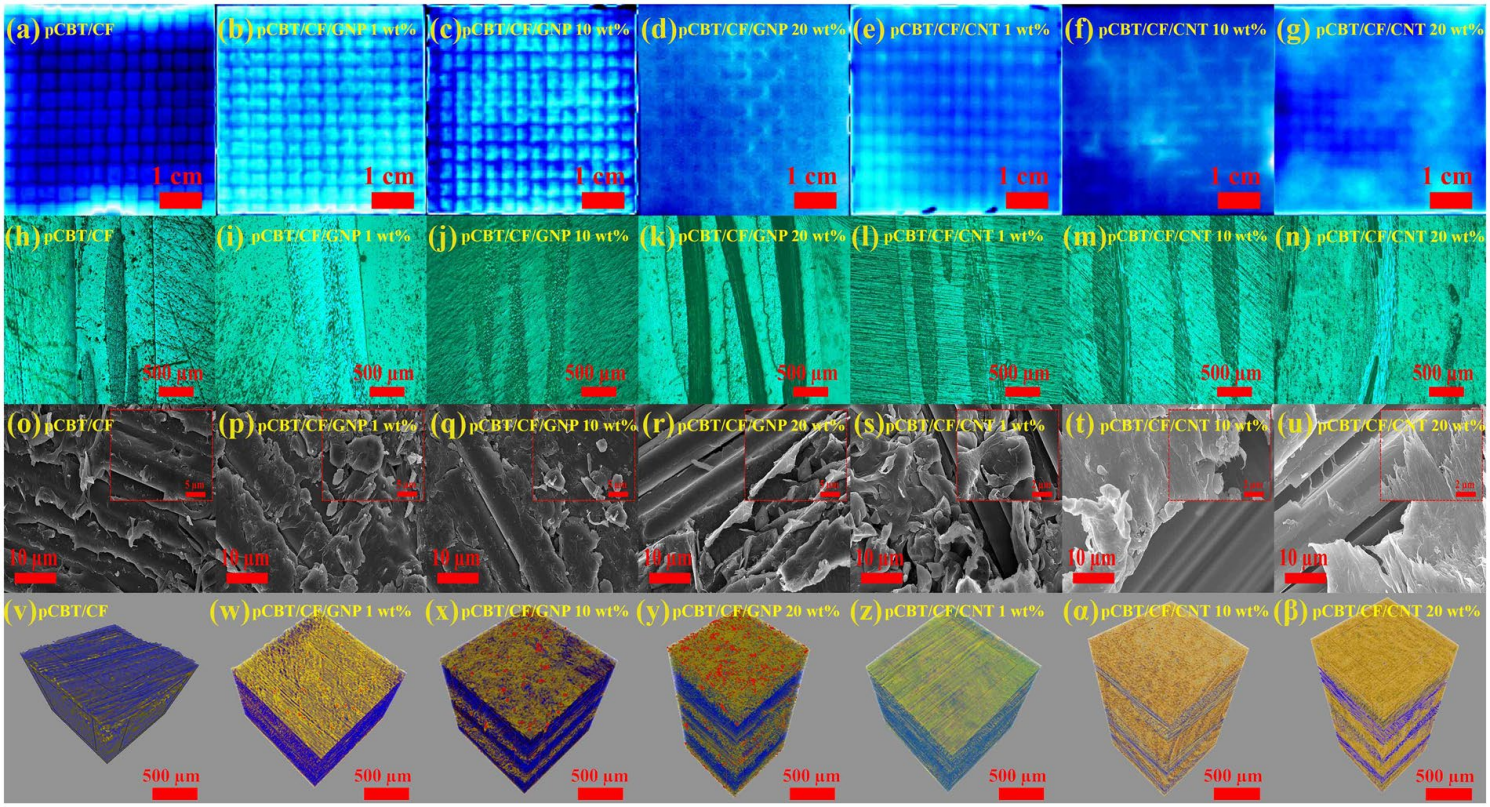
Active thermography images of CFRP composites (**a**) without nanocarbon fillers, with (**b**) 1 wt% GNP, (**c**) 10 wt% GNP, (**d**) 20 wt% GNP, (**e**) 1 wt% MWCNT, (**f**) 10 wt% MWCNT, (**g**) 20 wt% MWCNT, OM images of CFRP composites (**h**) without nanocarbon fillers, with (**i**) 1 wt% GNP, (**j**) 10 wt% GNP, (**k**) 20 wt% GNP, (**l**) 1 wt% MWCNT, (**m**) 10 wt% MWCNT, (**n**) 20 wt% MWCNT, FE-SEM images of CFRP composites (**o**) without nanocarbon fillers, with (**p**) 1 wt% GNP, (**q**) 10 wt% GNP, (**r**) 20 wt% GNP, (**s**) 1 wt% MWCNT, (**t**) 10 wt% MWCNT, (**u**) 20 wt% MWCNT, and micro-CT images of CFRP composites (**v**) without nanocarbon fillers, with (**w**) 1 wt% GNP, (**x**) 10 wt% GNP, (**y**) 20 wt% GNP, (**z**) 1 wt% MWCNT, (**α**) 10 wt% MWCNT, (**β**) 20 wt% MWCNT.

Despite the variations in the filler composition of multi-walled carbon nanotubes (MWCNTs) and graphene nanoplatelets (GNPs), as shown in the active thermography images displayed in Fig. 3a–g and Fig. S3a–f, pores larger than the system’s limiting resolution of 6 μm were not observed^19^. As shown in the OM images provided in Fig. 3h–n and Fig. S3g–l, CF fabric layers were clearly visible, and the CFs in the fabric maintained their original form, i.e., an array of intersecting horizontal and vertical lines. As shown in the FE-SEM images in Fig. 3o–u and Fig. S3m–r, the CFs were well aligned in the vertical direction and, except for the CF fabric, the space in the composite was fully occupied by the resin filled with carbon nanofillers. As shown in the micro-CT images in Fig. 3v–β and Fig. S3s–x, unfortunately, it was impossible to measure the dispersion of MWCNTs due to the system’s limited resolution (~0.7 μm)^19^; however, uniform dispersion of GNP fillers was found in the layers in which no CFs existed (see Supplementary video). Consequently, the three-component CFRP composites that were fabricated using the proposed process showed not only good impregnation of the CF fabric with the used matrix but also a uniform dispersion of nanofillers with few pores inside the composites.

**Figure 4:**
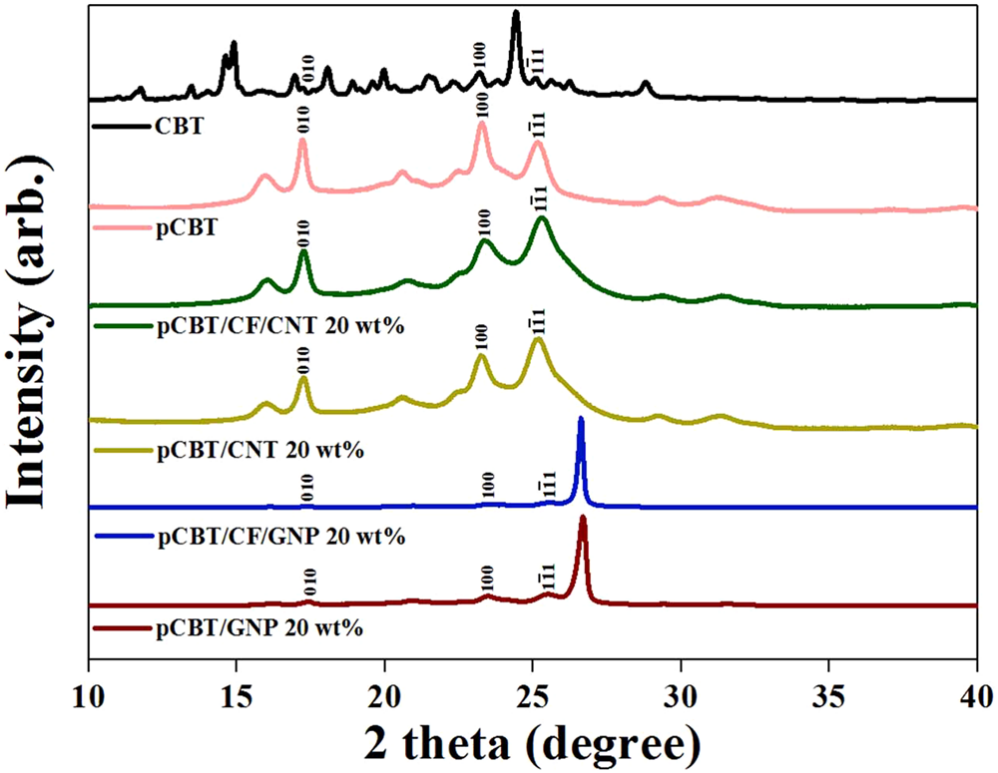
WAXD patterns of CBT oligomer and the pCBT composites filled with CFs and/or nanocarbon fillers.

Fourier transform infrared (FT-IR) spectra of the raw CBT matrix and the pCBT composites filled with CFs and/or nanocarbon fillers are shown in Fig. S4. Characteristic peaks of ester groups of the CBT matrix and pCBT composites occurred at 1714 cm^−1^ for C=O, at 1118 cm^−1^ for the C-O aliphatic end, and at 1103 cm^−1^ for the C-O aromatic end^20^. Unfortunately, it could not be determined from the FT-IR results if the CBT matrix was well polymerized during the composite fabrication process because the CBT resin exhibits the same functional groups before and after *in-situ* polymerization. When the CBT oligomer is polymerized to pCBT as a polymer, the crystallinity of the polymer matrix will appear. The irradiated X-rays could be scattered by the crystal structure of pCBT and appear as specific crystalline peaks in the X-ray diffraction characterization. Wide angle X-ray diffraction (WAXD) patterns of the CBT matrix and of the pCBT composites are shown in Fig. 4. The crystalline peaks observed for the CBT matrix showed that the CBT resin consisted of crystalline oligoesters. Except for the (002) diffraction peak of GNP at the Bragg angle of 27.5°, which makes the other peaks smaller in the WAXD patterns of GNP-filled pCBT composites^11^, the WAXD patterns of the pCBT matrix and composites were almost identical. The difference between the WAXD patterns of the CBT matrix and of the pCBT composites means that during the composite fabrication process the crystallization of the pCBT molecule occurred after *in-situ* polymerization of the CBT oligomer^21^. Therefore, it the three-component CFRP composites that were fabricated by the proposed processing method showed good impregnation of the CF fabric with pCBT molecules filled with uniformly dispersed nanofillers because the CBT molecules were polymerized to form pCBT molecules during the proposed process.

### Physical Properties

**Figure 5:**
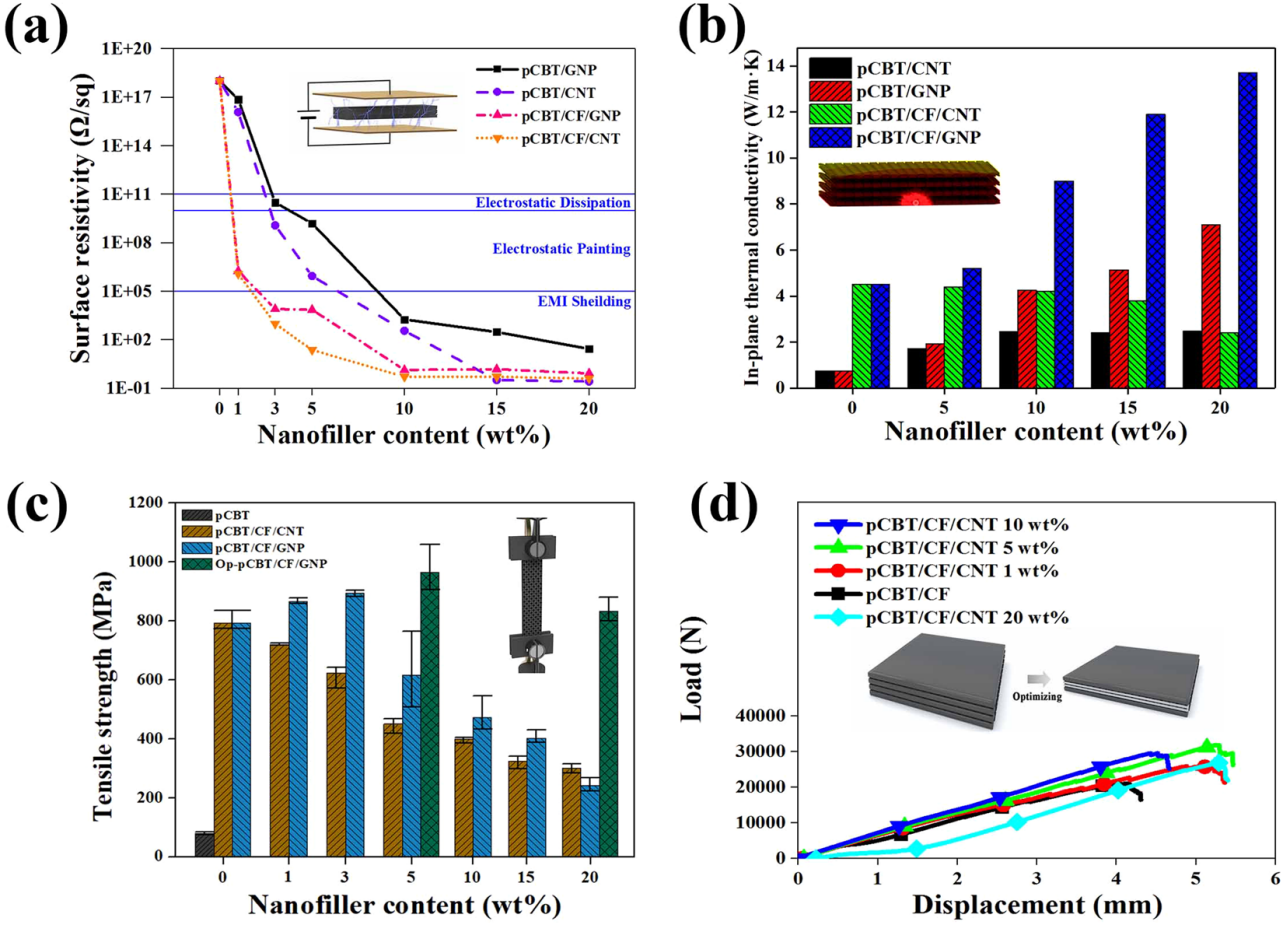
(**a**) Surface resistivity, (**b**) thermal conductivity, (**c**) tensile strength and (**d**) load-displacement curve of the pCBT composites filled with CFs and/or nanocarbon fillers.

Figure 5a shows the surface resistivity of the fabricated composites. In the two-component composites (consisting of nanofillers and the pCBT matrix) with the same nanofiller content, the surface resistivity of the composites filled with MWCNTs was lower than that of the composites filled with GNPs, indicating that the MWCNT is a more efficient filler than the GNPs for improving the electrical conductivity of the composites^13^. According to the percolation theory, it can be confirmed that the electrical conductivity was considerably enhanced as the path of the electrons was formed due to the nanofiller. The percolation threshold of the two-component composites was observed at 3 wt% of nanofiller content, whereas that of the three-component composites consisting of CF, nanofillers and the pCBT matrix was found at 1 wt% nanofillers. Interestingly, the difference between the surface resistivity of the three-component composites filled with MWCNTs and those filled with GNPs was slight. These results could be attributed to the fact that nanofillers were present within the tunnelling length of the electrons in the electron-rich CF layer, and the electrons of the CF could be transferred to the surface of the three-component composites. Therefore, the developed three-component composites can be utilized for applications requiring electrically conductive characteristics, such as electrostatic dissipation (<surface resistivity of 10^+11^ Ω/sq), electrostatic painting (<surface resistivity of 10^+10^ Ω/sq) and EMI shielding (<surface resistivity of 10^+5^ Ω/sq), by controlling the nanofiller content^22,23^.

Figure 5b shows the thermal conductivity of the fabricated pCBT composites. The three-component composites with MWCNTs achieved no obvious improvement in thermal conductivity, whereas the thermal conductivity of the GNP-filled three-component composites tended to increase linearly with increasing filler content. The GNP filler is considered suitable for improving the thermal conductivity of the composites in contrast to the MWCNT filler. Introducing nano-sized MWCNTs helps improve the thermal conductivity of the composites due to the high thermal conductivity of the fillers; however, these MWCNTs adversely affect the thermal conductivity by providing filler-resin interfaces. At the nanofiller-resin interfaces, incomplete contact and small contact areas result in an interfacial resistance, which is known to hinder phonon transport^24,25^. In particular, a CFRP composite filled with 20 wt% GNP showed the best thermal conductivity of 13.70 W/m·K, an improvement of 9033.3% and 93.0% compared to the values of the 0.15 W/m·K pCBT resin and of 7.10 W/m·K pCBT composites filled with 20 wt% GNP.

The tensile strength of the fabricated composites is shown in Fig. 5c. Due to the incorporation of CF, the tensile strength of the CFRP composite (794 MPa) was improved by 892.5% compared with that of the pCBT resin (80 MPa). This result indicates that the tensile strength of the CFRP composites was dominantly enhanced by the reinforcing CFs. With the additional introduction of nanofillers, the tensile strength significantly decreased at above a 5 wt% nanofiller content. As shown in the load-displacement graph in Fig. 5d, because the load level was slightly varied, the increased cross-sectional area of the specimen resulting from the incorporation of nanofillers can be a major factor that affects the decrease in the tensile strength of the three-component composites that have more than 5 wt% nanofillers. The cross-sectional area of the CFRP composites filled with GNPs with less than 5 wt% was not increased significantly, whereas that of the CFRP composites filled with bulky MWCNTs of less than 5 wt% was increased. Therefore, at a GNP content of less than 5 wt%, the tensile strength was additionally enhanced. Accordingly, there is a need to optimize the mechanical properties of high-content three-component composites, and adjusting the specimen thickness can be important for optimization.

### Tensile Property Optimization

Despite the application of identical processing conditions, it was not possible to equally control the specimen thickness due to the incorporation of bulky nanofillers. Fabricating a three-component composite filled with nanofiller to maintain its thickness at a level that is similar to that of the control could improve the tensile strength of the specimen to a level greater than that of the control. Considering the applications of the three-component composites, the conductive characteristics are more valuable in the in-plane direction than in the thickness direction. In this regard, we can propose a modification that adjusts the specimen thickness by controlling the composition of each layer so that the in-plane conductivity can be selectively enhanced. As shown in Fig. 5, when nanofillers were incorporated at 5 or 20 wt% only into the outermost layer, respectively, but were not contained inside, the three-component composites exhibited improvements in tensile strength and in their electrical and thermal conductivities. They were found to have conductive characteristics similar to those of the three-component composites filled with the 5 or 20 wt% nanofillers into both the outermost and inside layers, respectively. Therefore, the tensile properties of the three-component composites can be effectively controlled by adjusting the specimen thickness.

### Applications

**Figure 6:**
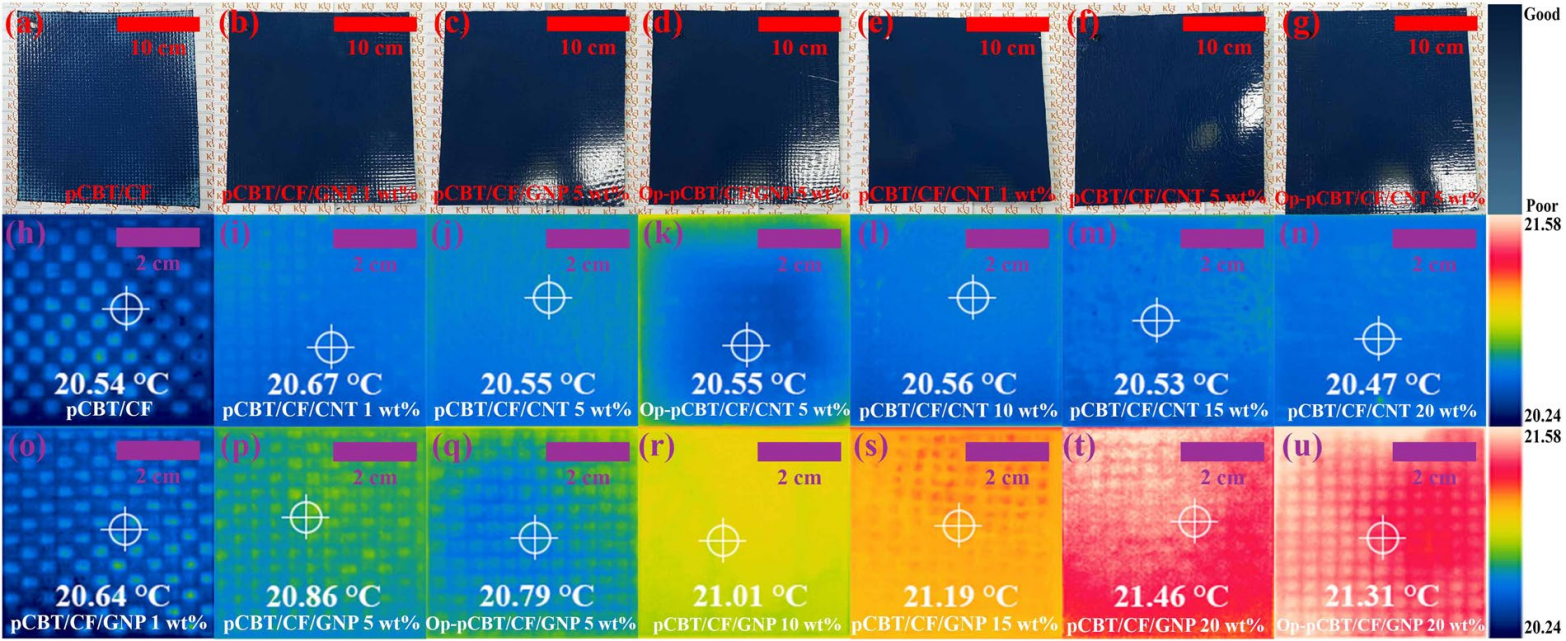
Electrostatic painting results of CFRP composites (**a**) without nanocarbon fillers, with (**b**) 1 wt% GNP, (**c**) 5 wt% GNP, (**d**) 5 wt% GNP only into the outermost layers, (**e**) 1 wt% MWCNT, (**f**) 5 wt% MWCNT, (**g**) 5 wt% MWCNT only into the outermost layers, infrared camera images of CFRP composites (**h**) without nanocarbon fillers, with (**i**) 1 wt% MWCNT, (**j**) 5 wt% MWCNT, (**k**) 5 wt% MWCNT only into the outermost layers, (**l**) 10 wt% MWCNT, (**m**) 15 wt% MWCNT, (**n**) 20 wt% MWCNT, (**o**) 1 wt% GNP, (**p**) 5 wt% GNP, (**q**) 5 wt% GNP into the outermost layers, (**r**) 10 wt% GNP, (**s**) 15 wt% GNP, (**t**) 20 wt% GNP, and (**u**) 20 wt% GNP only into the outermost layers.

Electrostatic powder painting is important for the use of composites in automotive outer panel parts. Figures 6a–g and S5a–c illustrate the results obtained from electrostatic powder painting of the fabricated CFRP composites (see Supplementary video). Previous studies reported that electrostatic powder painting can be applicable at an electrical conductivity on the order of ≥10^−5^ ^22,23^. Electrostatic powder painting was not successful in uniformly coating the CFRP specimen without nanofillers; however, uniform coating was observed when it was applied to nanofiller-containing CFRP specimens. Electrostatic powder painting could also be uniformly applied to the composites where nanofillers had only been introduced into the surface layer for optimization of the mechanical properties. These findings suggest that the proposed nanofiller-containing thermoplastic CFRP composites have potential applications in automotive outer panel parts.

Figure 6h–u shows the heat dissipation characteristics of the fabricated CFRP composites. Superior heat dissipation characteristics were observed for the composites showing high thermal conductivity; the tendency of thermal conductivity values relative to the composition of the composites was very consistent with the tendency of their heat dissipation characteristics. This study confirmed that optimizing the filler compositions and thermally conductive 3D filler networks is an important physical factor in optimizing the heat dissipation characteristics of composites and their thermal conductivities. It can, therefore, be concluded that the proposed nanofiller-containing thermoplastic CFRP composites have potential for application in automotive engine block parts and other components that require heat dissipation characteristics.

## Conclusions

To fabricate rapidly producible, repairable, and recyclable CFRP composites with excellent electrical and thermal conductivities, this paper proposes a new composite fabrication process that is based on powder mixing and *in-situ* polymerization of CBT oligomers. Because the CBT molecules were polymerized to form pCBT molecules during the proposed process, the thermoplastic CFRP composites filled with nanocarbon fillers that were fabricated using the proposed process showed good impregnation of CF fabric with pCBT molecules filled with uniformly dispersed nanofillers. The electrical conductivity of the three-component composites that consist of a CF, nanofillers and pCBT matrix was superior to that of two-component composites that consist of nanofillers and a matrix because nanofillers were present within the tunnelling length of the electrons in the electron-rich CF layer and because the electrons of the CF are transferred to the surface of the three-component composites. The GNP filler is considered suitable for improving the thermal conductivity of the composites in contrast to a MWCNT filler. The CFRP composite filled with 20 wt% GNPs showed the best thermal conductivity of 13.70 W/m·K. The tensile strength of the three-component composites that had nanofillers above 5 wt% was reduced due to the increased cross-sectional area of the specimen due to the incorporation of bulky nanofillers. The decrease in tensile strength can be overcome by controlling the thickness of the specimen by incorporating nanofillers only in the outermost layers of the three-component composites. Based on the applicability tests for electrostatic powder painting and heat dissipation, the nanofiller-containing thermoplastic CFRP composites have potential in automotive part applications, such as for outer panels and engine blocks, where electrostatic powder painting and/or heat dissipation characteristics are required.

## Methods

### Materials

GNPs (M25, XG Science, Lansing, MI, USA) and MWCNTs (CM-280, Hanwha Chemical Co., Seoul, Korea), which are known to be advantageous for enhancing the electrical and thermal conductivity of composites, were prepared as conductive fillers for the fabrication of conductive CFRP composites; CF fabrics (Hankuk Carbon Co., Ltd., Milyang, Gyeongnam, Korea) were used as reinforcement. The CFs constituting the fabrics were T-700 fibres with a density of 1.82 g/cm^3^. CBT (CBT 160, Cyclics® Co., Schenectady, NY, USA) is a low molecular weight ring-shaped oligomer that exists in a solid state at room temperature. The thermoplastic resin is ring-opened and melts at ≥150 °C; the molten oligomers are known to exhibit a low viscosity of 0.02 Pa·s. CBT molecules are polymerized by contained catalysts into a structure similar as that of poly(butylene terephthalate) when they are subjected to ≥170 °C^8^.

### Composite Fabrication

Enhanced dispersion of nanocarbon fillers can be induced by the low-melt viscosity of CBT molecules of 0.02 Pa·s during the initial thermal process^8,11,12^. For carbon fillers to remain highly dispersed, composites were fabricated using powder mixing, as shown in Fig. 1a. CBT powder and nanocarbon fillers were mixed to the target content using a Thinky mixer (ARE 310, Thinky Co., Tokyo, Japan) for 1 min at 2000 rpm and for another 1 min at 2200 rpm. The prepared mixture was sufficiently sprayed onto a metallic mould; then, a CF fabric was laminated onto it. The process was repeated to obtain the intended thickness of the specimen, as shown in Fig. 1b. Three-component composites were fabricated by compressing the layers of lamination using a heating press (D3P-20J, Dae Heung Science, Incheon, Korea) with the metallic mould heated to 250 °C at 15 MPa for 2 min, as shown in Fig. 1c. The composition of the fabricated CFRP composites is summarized in Table 1. In addition, we prepared various control samples, including a polymerized CBT (pCBT) specimen, a CFRP specimen without nanocarbon fillers (see Fig. S1), and pCBT composites filled with nanocarbon fillers (see Fig. S2), using a previously proposed fabrication method^11–13,15,17,18^.

**Table 1:** Composition of the fabricated CFRP composites.

	Weight fraction (%)		
	CF	CBT matrix	Nanocarbon
pCBT/CF	50	50	0
pCBT/CF/GNP 1 wt%	50	49.5	0.5
pCBT/CF/GNP 3 wt%	50	48.5	1.5
pCBT/CF/GNP 5 wt%	50	47.5	2.5
Op*-pCBT/CF/GNP 5 wt%	50	48.75	1.25
pCBT/CF/GNP 10 wt%	50	45	5
pCBT/CF/GNP 15 wt%	50	42.5	7.5
pCBT/CF/GNP 20 wt%	50	40	10
Op-pCBT/CF/GNP 20 wt%	50	45	5
pCBT/CF/CNT 1 wt%	50	49.5	0.5
pCBT/CF/CNT 3 wt%	50	48.5	1.5
pCBT/CF/CNT 5 wt%	50	47.5	2.5
Op-pCBT/CF/CNT 5 wt%	50	48.75	1.25
pCBT/CF/CNT 10 wt%	50	45	5
pCBT/CF/CNT 15 wt%	50	42.5	7.5
pCBT/CF/CNT 20 wt%	50	40	10

*Nanofillers were incorporated only into the outermost layer.

### Internal Structure Characterization

First, we performed typical internal structure analyses, such as OM and FE-SEM. These are tools for obtaining two-dimensional images that are capable of assessing the surface pores, filler dispersion and resin impregnation of composites; however, these tools cannot cover the entire internal structure of the materials because they are limited to analyses of the locally imaged regions. Pores for the entire area of the specimens were analysed using a non-destructive analysis system called active thermography. In addition, precise structural information on three-dimensional (3D) nanofiller dispersion, CF fabric impregnation, CF orientation and micro-sized pore/defects in the composites was obtained using micro-CT, which is a non-destructive 3D analysis technique.

The fabricated composite specimens were polished using a polishing machine (TegraPol-15, Struers, Ballerup, Denmark). The fracture surface of each composite was observed with an OM (BX51, Olympus Co., Tokyo, Japan). Furthermore, the polished fracture surface was observed at 15 kV with a FE-SEM (Nova NanoSEM 450, FEI Co., Hillsboro, OR, USA) after being coated with platinum in a vacuum for 200 sec using a sputter coating machine (Ion Sputter E-1030, Hitachi High Technologies Co., Tokyo, Japan). The active thermography measurement was based on an infrared camera (X6540sc, FLIR systems, Wilsonville, OR, USA) that was utilized to observe internal defects (>6 μm)^19^. The prepared specimens (50 mm × 50 mm size) were heated using radiation emitted from a halogen lamp, and the lock-in thermography technique was used to convert the temperature distribution to an image. Micro-CT (Skyscan 1172, Bruker Co., Billerica, MA, USA) was used to measure and identify the 3D morphology, dispersion and network structure of the nanocarbon fillers in pCBT composites filled with CF and nanocarbon fillers. The detailed procedures for measuring micro-CT analysis are explained in Fig. S6. FT-IR (Nicolet 6700, Thermo Scientific, Waltham, MA, USA) was used to analyse the functional groups of the raw CBT matrix and the pCBT composites filled with CF and/or nanocarbon fillers. FT-IR spectra were measured in the range of 500–4000 cm^−1^ at a resolution of 16 cm^−1^. WAXD measurements were carried out using an X-ray diffractometer (M18XHF-SRA, MAC Science Co., Yokohama, Japan) with Ni-filtered, CuKα X-rays (λ = 0.1542 nm) to investigate the crystalline structure of the CBT matrix and the pCBT composites. The diffraction intensity was recorded by continuous scanning at a rate of 0.02 deg/s over a range of 10 < 2θ < 40 deg (θ = Bragg angle).

### Physical Property Measurements

The surface resistivity of the pCBT composites was measured using the four-probe method according to ASTM D257 (FPP-RS8, DASOL ENG, Cheongju, Korea) and an ultrahigh resistance meter (SM-8220, HIOKI E. E. Co., Nagano, Japan) under an applied voltage of 10 V. The thermal conductivity of the composites was measured under ambient temperature and pressure conditions using a TPS 2500 S instrument (Hot Disk AB, Gothenburg, Sweden), which is based on the hot disk method following the ISO 22007-2 standard. The hot disk sensor is made of a double spiral of thin nickel wire and works as a continuous plane heat source. The hot disk sensor supplies a constant amount of electrical power (P), which causes a temperature rise (ΔT) that can be measured according to changes in the sensor resistance. From the values of P and ΔT, the thermal conductivity (λ) can be calculated by solving the Fourier equation for heat conduction. The mechanical properties of the composite specimens were measured according to the ASTM D3039 standard using a universal testing machine (Instron 5982, Instron Co., Norwood, MD, USA) at a crosshead speed of 5 mm/min at room temperature.

### Tensile Strength Optimization

As shown in the inset image of Fig. 5d, the specimen thickness can be reduced by incorporating nanofillers into the surface but not the interior of the. The optimized specimen was fabricated using the same process except for the difference between the nanofillers-incorporated layer and non-incorporated layer.

### Application

In general, CFRP composites applied to automotive outer panels can be painted by electrostatic powder painting process. The fabricated CFRP composite specimens of 250 mm × 250 mm size were placed on a conveyor line, and a negative direct current (DC) voltage was passed through them. Then, the powder was sprayed through the spray device with a positive DC voltage. After the sprayed powder adhered to the specimen by electrostatic force, the powder melted and was coated on the surface of the specimen by applying heat at 200 °C for 10 min. The heat dissipation property of the pCBT composites was evaluated using an infrared camera (X6540sc, FLIR systems, Wilsonville, OR, USA) after heating the specimens for 4 sec using radiation emitted from a halogen lamp.

## Electronic supplementary material

Supplementary information

Supplementary video
