# Supplementary material for: Electrically and Thermally Conductive Carbon Fibre Fabric Reinforced Polymer Composites Based on Nanocarbons and an In-situ Polymerizable Cyclic Oligoester
Source: Sci Rep. 2018 May 16;8:7659. doi: 10.1038/s41598-018-25965-w (PMC5955969; doi:10.1038/s41598-018-25965-w)
Supplement: Supplementary file 1 — Supplementary information [file 41598_2018_25965_MOESM1_ESM.doc]

Electrically and Thermally Conductive Carbon Fibre Fabric Reinforced Polymer Composites Based on Nanocarbons and an In-situ Polymerizable Cyclic Oligoester

Ji-un Jang^1,2^, Hyeong Cheol Park^1^, Hun Su Lee^1^, Myung-Seob Khil^2^, Seong Yun Kim^2^,*[[1]](#footnote-2)


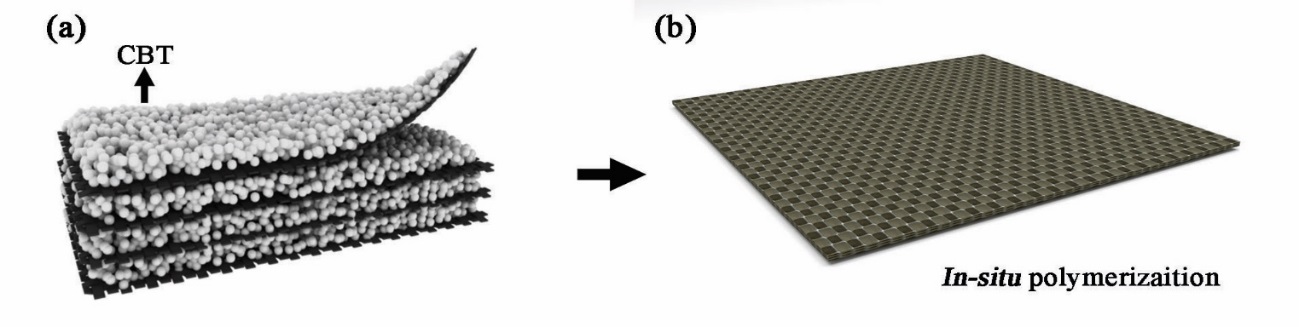


**Figure S1.** Schematic of fabrication process for fabricating a thermoplastic CFRP composite specimen without nanocarbon fillers.


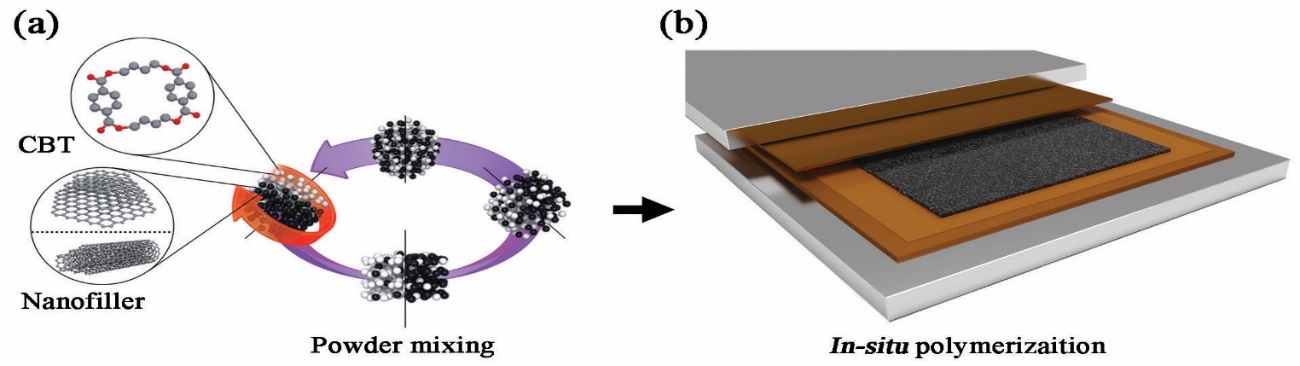


**Figure S2.** Schematic of fabrication process for fabricating pCBT composites filled with nanocarbon fillers.


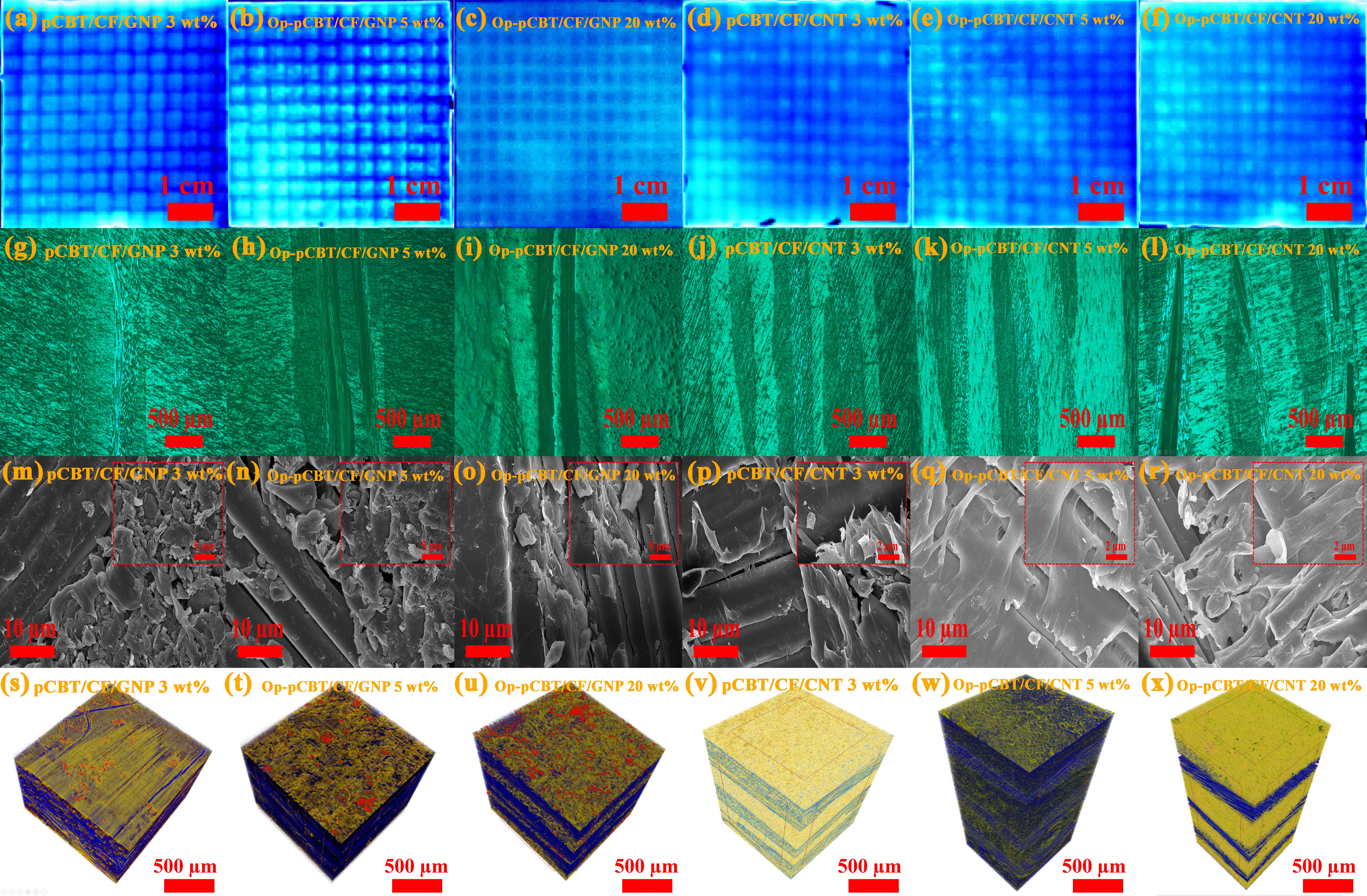


**Figure S3.** Active thermography images of CFRP composites with (a) 3 wt% GNP, (b) 5 wt% GNP only into the outermost layers, (c) 20 wt% GNP only into the outermost layers, (d) 3 wt% MWCNT, (e) 5 wt% MWCNT, (f) 5 wt% MWCNT only into the outermost layers, OM images of CFRP composites with (g) 3 wt% GNP, (h) 5 wt% GNP, (i) 15 wt% GNP, (j) 1 wt% MWCNT, (k) 5 wt% MWCNT, (l) 15 wt% MWCNT, FE-SEM images of CFRP composites with (m) 3 wt% GNP, (n) 5 wt% GNP, (o) 15 wt% GNP, (p) 3 wt% MWCNT, (q) 5 wt% MWCNT, (r) 15 wt% MWCNT, and micro-CT images of CFRP composites with (s) 3 wt% GNP, (t) 5 wt% GNP, (u) 15 wt% GNP, (v) 3 wt% MWCNT, (w) 5 wt% MWCNT, (x) 15 wt% MWCNT.


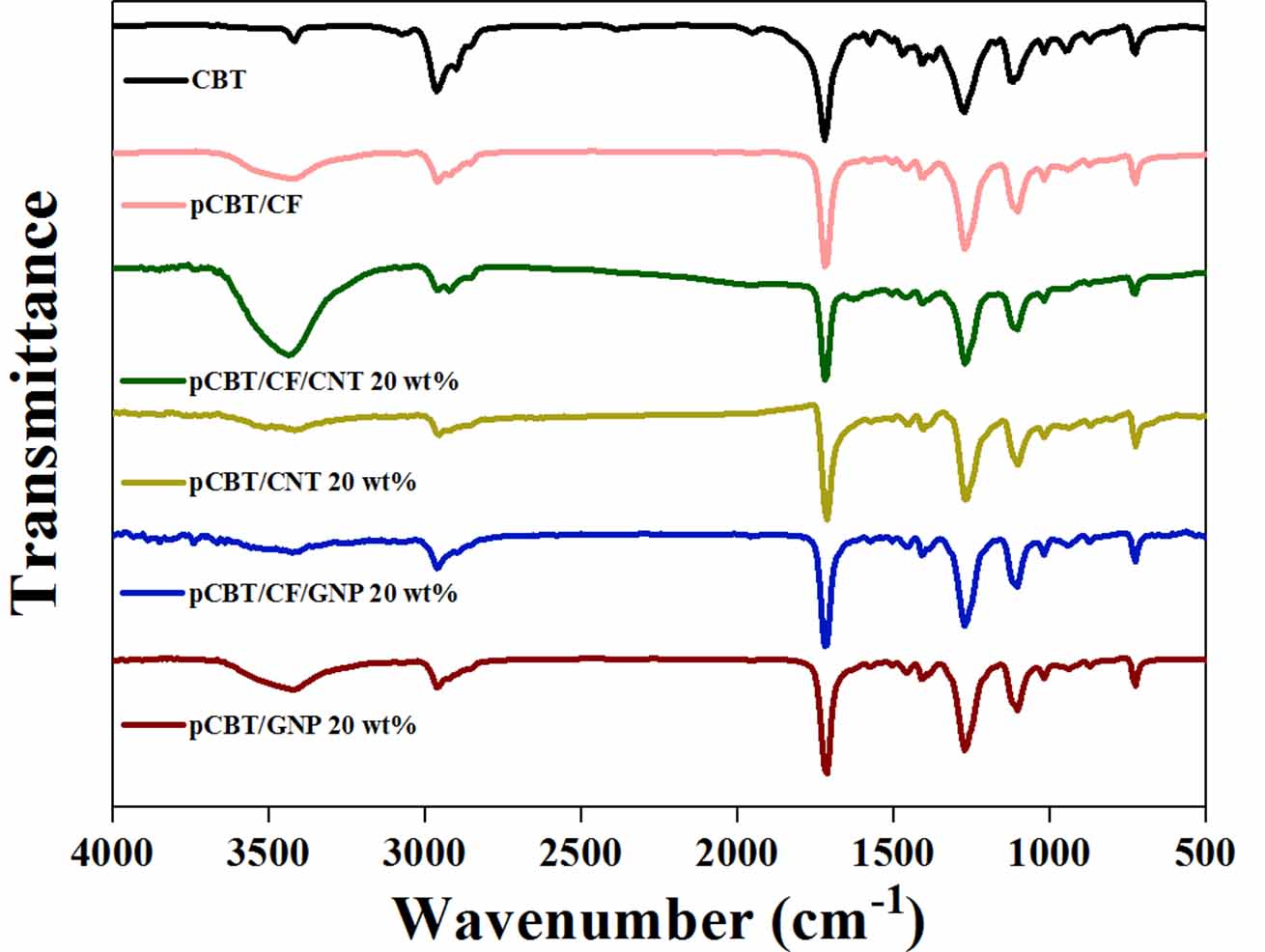


**Figure S4.** FT-IR spectra of CBT oligomer and the pCBT composites filled with CFs and/or nanocarbon fillers.


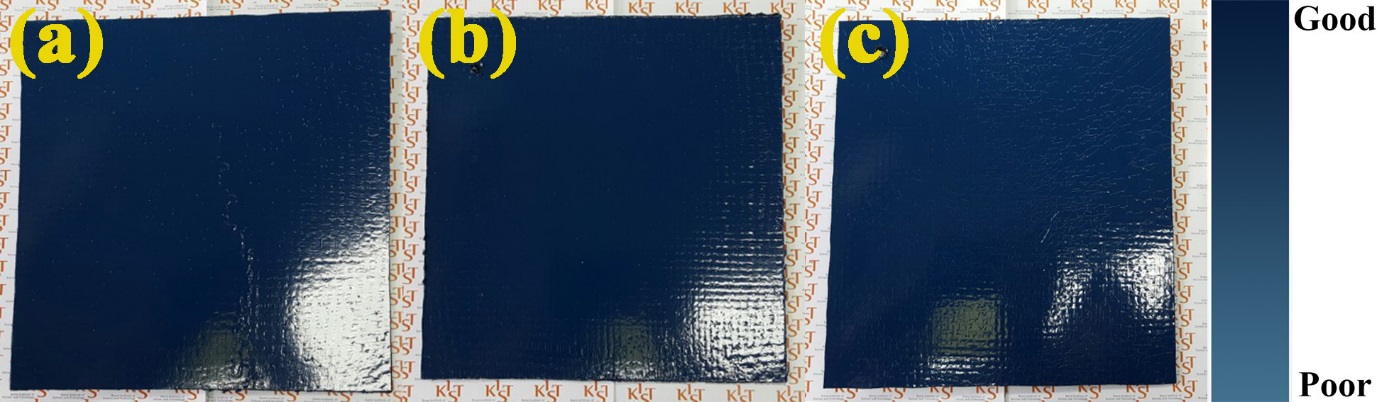


**Figure S5.** Electrostatic painting results of CFRP composites with (a) 10 wt% GNP, (b) 20 wt% GNP and (c) 3 wt% MWCNT.

**
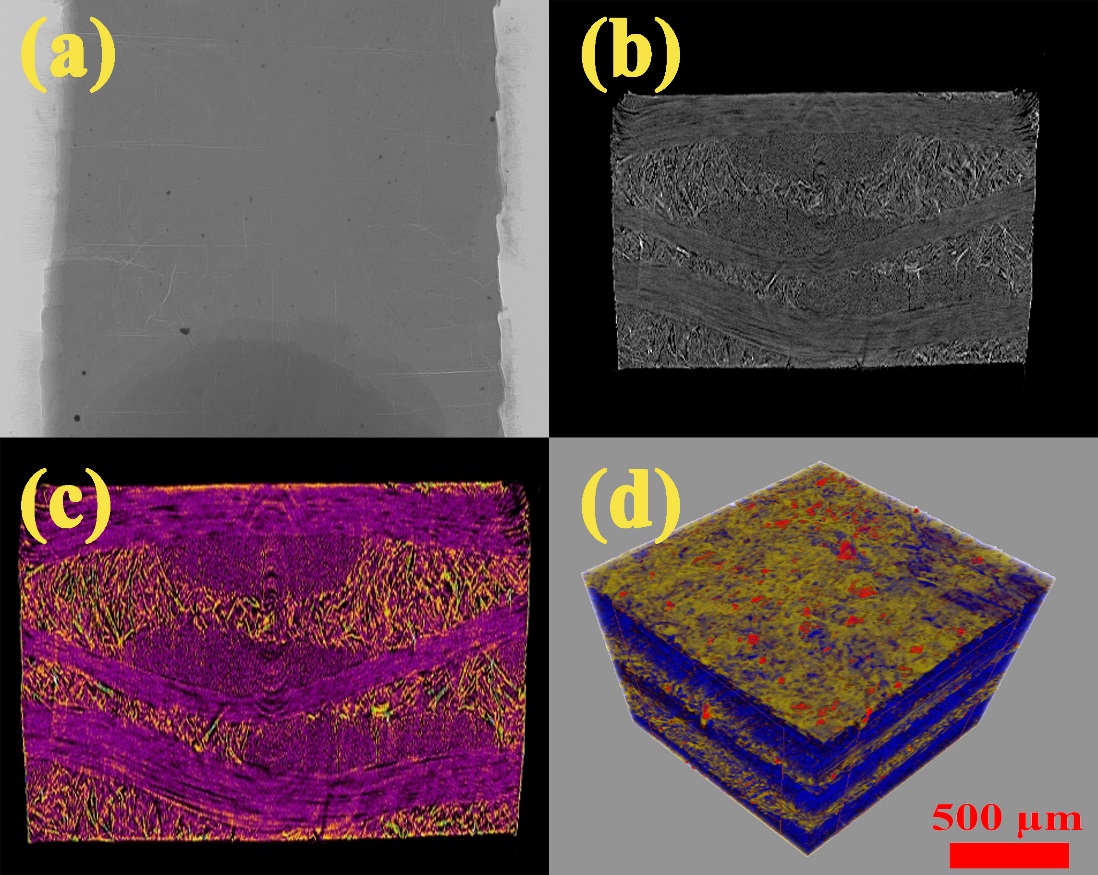
**

**Figure S6.** Micro-CT 2D images (a) before applying functions, (b) applied reconstruction function, (c) applied thresholding function, and (d) micro-CT 3D image applied 3D building function.

The raw image (Fig. S6a) measured by micro-CT can be reconstructed as a tomographic image, as shown in Fig. S6b. In order to distinguish CF, nanofiller and matrix, a thresholding function can be applied to the reconstructed image, as shown in shown in Fig. S6c. Finally, 3D micro-CT images can be generated using the 3D building function, as shown in Fig. S6d.

1. ^1^Mutifunctional?Structural Composite Research Centre, Institute of Advanced Composite Materials, Korea Institute of Science and Technology (KIST), 92 Chudong-ro, Bongdong-eup, Wanju-gun, Jeonbuk, 55324, Republic of Korea.

   ^2^Department of Organic Materials and Fiber Engineering, Chonbuk National University, 567 Baekje-daero, Jeonju-si, Jeonbuk, 54896, Republic of Korea

   *Correspondence and requests for materials should be addressed to S.Y.K. (email: sykim82@jbnu.ac.kr) [↑](#footnote-ref-2)
